# The Colleges of the Central West

**Published:** 1898-08

**Authors:** 


### THE COLLEGES OF THE CENTRAL WEST.

THE colleges of the Central West are some four in number. The older one, at Ames, Iowa, in connection with the Agricultural College, with a curriculum of three years of eight months each, has the longest course of study.

The Kansas City is the next in length of years, and maintains a course of three years of six months each.

The Indiana Veterinary College, at Indianapolis, maintains a two-years' obligatory course, with an optional third-year attendance. The many rapid changes in the corps of instructors tend to lessen the value of the teaching.

The Western Veterinary College, of Kansas City, Mo., is a new school. It proposed in its initial prospectus to maintain a curriculum in accord with the requirements of the Association of Faculties and the U. S. V. M. A. But the first year of existence caused its relinquishment of this plan and the adoption of a short two-years' course. It has also adopted a "stockman's" course, a very dangerous precedent, and one that will be more forcibly appreciated in the event of future legislation tending to define and regulate the requirements for the practice of veterinary science.

These schools have a wide field for new students, and no doubt their classes will pick up much greater this year than last.

